# Axillary Dissection in Breast Cancer Patients with Metastatic Sentinel Node: To Do or Not to Do? Suggestions from Our Series

**DOI:** 10.5402/2011/527904

**Published:** 2011-08-02

**Authors:** M. Bortolini, F. Genta, Chiara Perono Biacchiardi, E. Zanon, M. Camanni, F. Deltetto

**Affiliations:** ^1^Ginteam, Mini-Invasive Gynaecological and Breast Unit, Evangelical Hospital, ASL TO1, Corso Marconi 35, 10125 Torino, Italy; ^2^Breast Radiology Unit, Evangelical Hospital, ASL TO1, Torino, Italy

## Abstract

Several studies have put to question and evaluated the indication and prognosis of sentinel lymph node biopsy (SNLB) as sole treatment in human breast cancer. We reviewed 1588 patients who underwent axillary surgery. In 239 patients, axillary lymph node dissection (ALND) was performed following positive fine needle aspiration cytology (FNAC), and, in 299 cases, ALND was executed after positive SNLB. The most dramatic result from our data is that patients with either micrometastasis of the sentinel lymph node (SLN) or only metastatic SLN have, respectively, an 84.5% and a 75.0% chance of having no other nodal involvement. We believe a more refined patient selection is neccessary when considering ALND. Where the primary tumor is larger than 5 cm, where radio or adjuvant therapies are not indicated, in cases of FNAC+ nodes, and in cases presenting more than one metastatic sentinel node, we prefer to carry out ALND. Having thus said, however, our data suggests that it is wise not to perform ALND in almost all cases presenting positive SLNs.

## 1. Introduction

In 1994, Giuliano and colleagues published the original study concerning the feasibility and accuracy of intraoperative sentinel lymph node biopsy (SLNB) [1]. Today, SLNB is a widely accepted technique in the management of breast cancer in patients with clinically negative axilla. 

The literature in merit has since reported that less than half of all patients who have undergone axillary lymph node dissection (ALND) following a positive SLNB have shown additional metastases [2]. Negative ALND patients did not receive any therapeutic benefit from the more invasive procedure. In micrometastatic SLN patients, the frequency of non-SLN metastasis is lower. ALND often requires a second surgical intervention and signifies marked comorbidity, including lymphedema, arm paresthesia, chronic pain, and a decrease in arm and shoulder function in 5 to 50% of patients. In contrast, SLNB has a much lower rate of complications [3].

Current guidelines from the National Comprehensive Cancer Network (NCCN) [4] and the American Society of Clinical Oncology (ASCO) [5] recommend ALND for patients who have SLN metastases >0,2 mm. Only the NCCN addresses the possibility of avoiding ALND in patients with favorable tumors or in patients destined to adjuvant therapy when affected by serious comorbidity conditions.

Veronesi published a study involving 516 breast cancer patients treated with breast conserving surgery randomized in two study groups: SLNB followed by ALND and SLNB followed by ALND only when metastatic SLN were found. In the first group, 5% of patients with negative SLN were found to have a false-negative SLN. In the second group, the same rate of recurrence was expected. The observed cumulative incidence of axillary recurrences in the second group who did not receive ALND was found to be lower than 5% [6].

Several recent studies have suggested avoiding ALND in selected patients [7, 8]. In 40 to 60% of patients, the SLN is the only positive lymph node [9]. Several groups use nomograms to assess the risk of nonsentinel node involvement in these patients [10], while other investigators consider axillary dissection necessary to ensure adequate locoregional control [11].

The American College of Surgeons Oncology Group Z0011 Randomized Trial included 891 patients and compared locoregional recurrences and overall survival in SLN-positive patients who did or did not undergo ALND [12]. They demonstrated that the locoregional recurrence rates are not improved by ALND after SLNB. They also reported that ALND is not associated with improved survival in patients with micro- or macrometastases in the SLNs.

Another substantial question is the postoperative treatment planning. The AMAROS trial showed that the absence of knowledge regarding the extent of nodal involvement appears to have no major impact on the administration of adjuvant therapy [13].

Other investigators note that approximately 50% of patients have nonsentinel node metastases and are favourable to ALND which provides additional prognostic information and decreases locoregional recurrences. Few studies found additional positive nodes and distant recurrences in breast cancer patients with micrometastasis in SLN and suggest that ALND with adjuvant therapy should be considered [14, 15].

The aim of this study is to determine, in our series, which patients could have avoided ALND in cases of metastatic sentinel lymph nodes. We underline that clinical evaluation of axilla by ultrasounds (USs) and fine needle aspiration cytology (FNAC) was not the method of choice in most cases.

## 2. Patients and Methods

We conducted a retrospective review of 2180 cases of female patients who had undergone breast cancer treatment in our center between January 1, 2003, and February 28, 2011. We excluded 592 records: 120 patients who had undergone neoadjuvant therapy, 83 recurrences, 260 patients affected by carcinoma in situ of the breast, 48 metastatic patients, and 81 “cup syndrome” patients or older patients who did not receive any kind of axillary surgery. 1588 patients remained eligible for the study.

SLNB was carried out in patients negative in both clinical and ultrasound axilla examination. In suspect cases, FNAC was performed to assess the lymph node status. 

SLNB was performed by means of an isotope injection with intraoperative detection via gamma probe. Lymphoscintigrams were executed in every patient. In cases of radioactive node detection failure, a periareolar injection of patent blue dye was performed. All stained or radioactive lymph nodes were removed.

In most cases of positive micro- or macrometastatic SLN, patients underwent ALND. Where SLN positivity was described as isolated tumor cells (ITCs), ALND was not considered. Most patients underwent ALND as a second, delayed intervention. In cases of stain and radioactive detection failure, immediate ALND was performed.

ALND denotes removal of the 1st and the 2nd levels of axillary nodes. The 3rd nodal level is removed only when clinically pathologic nodes are identified in the second level.

Patient records were fully documented in a proprietary database which included registration of all data regarding intervention, preoperative and postoperative histologic diagnosis, number and status of lymph nodes removed, pathologic TNM, and followup. Statistical distribution and Pearson's Chi-squared test were performed by EpiInfo software.

## 3. Results

To remove the primary tumor, a breast-conserving procedure was performed in 75.3% of patients and mastectomy was performed on the remaining 24.7% of patients. Choice of procedure was decided preoperatively based on clinical grounds, and tumor specimens underwent routine laboratory pathological study.

Mean patient age was 60 years old, and median follow-up time was 38 months. 

A mean number of 1.3 SLNs were removed during SLNB, and a mean number of 18.2 lymph nodes were removed per ALND. Sentinel node detection rate in our series was 96.28%, combining radioactive and staining techniques. 57 patients were operated before 2005, when ASCO Guidelines [6] suggested immediately ALND in breast cancer patients with a presenting tumor greater than 3 cm. In 48 patients, the SLN was not identified, and in 40 of these patients ALND was performed. 

In 239 of the remaining 1483 women (16,1%), ultrasound axilla and FNAC showed metastatic disease. All underwent immediate axillary dissection. In this set of patients, we recorded 2 (0.8%) patients with pN1mi, 117 (49%) patients with pN1a, and 120 (50,2%) with pN2a or pN3a (Table 1). 

We reviewed a total of 1244 patients that underwent ALND, recording frequencies of 3,7% of pT1mic (46), 5,9% of pT1a (73), 20,4% of pT1b (254), 44,5% of pT1c (554), 22,7% of pT2 (282), and 2,8% of pT3 and pT4 (35) combined (Table 2).

Of the 260 pTis patients, 116 SLNBs were executed yielding 107 negative results, 5 not identified SLN, and 4 ITC cases. None underwent ALND.

Of the 1244 SLNB patients, 299 (24%) underwent delayed axillary dissection following diagnosis of sentinel node metastases, 861 resulted negative SLN, and 84 SNLB positive for metastatic disease stopped their surgical path (60 ITC, 20 micrometastases, and 4 macrometastases).

115 patients presented micrometastases in their SLN, and 208 patients presented metastases greater than 2 mm. In 80/95 patients after ALND stage pN1mi was confirmed, (84.2%), and 11/95 (11,6%) presented a definitive pN1a. Only 4 patients had a definitive pN2a or pN3a. pN1a(sn) patients presented 75% (153/204) of pN1a after ALND, 17,2% (35/204) of pN2a, and 7,8% (16/204) of pN3a (Table 1).

In 55 cases where multiple SLNBs were removed, we noticed a higher probability to have more severe definitive staging with multiple positive SNLs (48,2%, 29/55; *P* < 0.0001) (Table 3).

Since FNAC is less sensitive than SLNB, FNAC selects more advanced disease. In fact we noted a higher lymph nodes involvement among the 239 FNAC selected patients (Table 1).

927 patients presented mass of tumor <2 cm, 77 of these (8,3%) in resulted micrometastasis at SLNB. We noted 100 macrometastases in pT1 cases (10,1%).

In 282 patients with pT2 staging where SLNB was executed, we found 37 patients with micrometastases (13,1%) and 90 pN1a (sn) patients (31,9%). 

In 35 pT3 and pT4 patients, we found 1 micrometastasis (2.9%) and 18 macrometastases (51,4%) (Table 2). The direct relationship between poor lymph nodal status and higher pT is obvious.

In 99 patients with pT1 and pN1a(sn) and 89 patients with pT2 and pN1a(sn), all of whom underwent ALND, we noted a higher incidence of patients with pT2 lobular cancer to have multiple positive SLNs versus patients with ductal cancer (*P* < 0.005) (Table 4).

Following median followup of 38 months, we observed 9 axillary recurrences alone (ARA), 4 axillary recurrences within regional recurrences (ARR), and 4 axillary recurrences within metastatic disease (ARM).

635 patients underwent ALND for varying therapeutic strategies, none evidenced ARR, and 3 presented ARM.

Of the 861 negative SLN patients, we observed 14 with recurrences (1,6%): 8 ARA, 3 ARR and 3 ARM (Table 5).

## 4. Discussion

SLNB for axillary staging has saved many breast cancer patients from ALND and resultant complication, when the SLN was found to be negative for metastatic disease. Knowing when to limit ALND is the objective of our discussion.

Recent data suggests avoiding ALND in cases of positive SLN where treatment strategy includes whole breast radiotherapy alone or combined with adjuvant therapy following conservative surgery of T1-2 breast cancer.

Here, we open a wide area of discussion [8, 10].

Not indicating ALND in the forementioned patients would by itself yield a mathematical advantage in patient quality of life, but a fine tuning selection of subgroups with differing axillary involvement could be worth study, as differing rates of axillary recurrence and mortality are expected.

Only clinically negative axillary patients are eligible for SLNB. In most published studies, “clinical staging” consists in clinical examination alone [16]. Today, both US and FNAC are routinely used for preoperative evaluation of axilla [17]. The combined technologies are producing a selection of FNAC-positive patients, who no longer need SLNB.

Our data shows that of the 239 patients that underwent ALND following an FNAC positive SLN, a significantly higher frequency of axillary involvement (pN2a-3a) (120/239, 50.2%) was observed in respect to FNAC-negative patients where delayed ALND was performed as direct segue to positive SLNB (55/299, 18.4%, *P* < 0.0001). 

If we consider FNAC-positive patients as clinically positive, they become eligible for ALND. 

We would otherwise be accepting a risk of leaving consistent residual axillary disease in 50% of the cases. it may be in these cases where the literature shows that the theoretical risk does not manifest recurrence.

Several conditions could explain such low rates of recurrence: immunologic systemic surveillance, subsequent radio and adjuvant therapy, and lack of stem cells in axillary lymph nodes to support cancer progression. The hypothetical risk, however, could be higher in these patients [9, 18, 19].

Residual axillary disease in cases of positive SLNB that could spare ALND would be only 18.4%. The use of published nomograms [10] could work to further select subgroups of risk, rendering the choice to withhold ALND easier.

Though our data evidenced increased lymph node involvement directly related to tumor size, where tumors range between 1 (pT1c) and 5 centimetres (pT2), the rates of lymph node involvement are not indicative in the selection of patients who can safely avoid ALND (*P* < 0.0001, Table 2).

While lobular type was associated with a greater nodal involvement in case of T2 cancers, no histological variable had significant prognostic power in the selection of ALND candidates (*P* < 0.005, Table 4).

In cases of micrometastatic SLN it is evident, and our data confirms the literature in merit, that nodal involvement is minimal to the point that omission of ALND is obvious [20]. 

In our complete series we recorded 20 axillary recurrences in 1588 patients. Three were part of the 635 patients that underwent ALND, each occurring in metastatic patients, the remaining 17 axillary recurrences occurred among the 953 non-ALND patients. 14 of 861 were negative SLNB, and 3 of 60 were SNLB-positive ITC.

It is worthwhile to consider that the omitted dissection of the third nodal level does not imply an augmented risk of recurrence.

In 115 micrometastatic SLNB patients (20 without ALND) no axillary recurrences were recorded. 

Since we accept the omission of 5% of positive non-SLNs where SLNB is performed, we can accept both the similar omission of 4.2% (4/179) in case of SLNs positive for ITC or micrometastases and the worse condition in case of macrometastasis in SLN: 51/204 (25%). 

Where we found more than one metastatic SLN, we observed after ALND 14/29 cases classified as pN2a and pN3a (48,2%), findings very similar to FNAC-positive patients. These patients should be candidates for ALND.

The last subject of discussion is adjuvant therapy. The AMAROS study [13] showed that knowledge of axillary status did not modify postoperative treatment planning. Are we sure that the same treatment would be chosen by all oncologists and radiotherapists in so varying conditions like positive SLNB and pN2a-3a after ALND [21]?

Do they all agree on extended fields of radiotherapy and on taxanes administration for the patient presenting one single positive SLN?

## 5. Conclusions

While continuing to perform ALND in cases of mastectomy, nonplanned whole breast radiotherapy, non planned adjuvant therapy, tumors larger than 5 cm, FNAC-positive nodes, and multiple positive SLNs, we propose a more prudential selection of the remaining ALND candidates. 

We suggest avoiding ALND in cases of micrometastatic SLN and in all remaining positive SLNs. 

Our data shows doing so would safely avoid a significant number of ALNDs. 

We executed 578 ALNDs: 239 were FNAC+, 40 not identified SLNs, 95 micrometastatic SLNs, and 204 were macrometastatic SLNs on 136 breast conservative treatments and 68 mastectomies. Twenty-nine presented multiple positive SLNs: 11 of these underwent concomitant breast conservative treatment and 18 with concomitant mastectomy. 

In retrospection, we could have avoided ALND in 95 micrometastatic SLNs and in 125 macrometastatic nonmultiple SLNs with concomitant breast conservative treatment. 

This translates to an sparing of 38% (220/578) among all ALNDs or an avoidance of 64.8% (220/339) among SLNBs, with a concomitant risk of 12.3% (27/220) of not surgically treating residual axillary disease classified as pN2a or pN3a.

## Figures and Tables

**Table 1:** Pathologic findings after ALND in case of FNAC or SNB positivity (we have higher lymph nodes involvement if FNAC is positive; *P* < 0.0001).

pN	FNAC + (%)	SLN + All (%)	pN1mi (sn) (%)	pN1a (sn) (%)
1mi	2	80	80	—
	*0,8*	*26,8*	*84,2*	—
1°	117	164	11	153
	*49*	*54,8*	*11,6*	*75*
2a	73	38	3	35
	*30,5*	*12,7*	*3,2*	*17.2*
3a	47	17	1	16
	*19,7*	*5,7*	*1*	*7,8*

Total	239	299	95	204

**Table 2:** SLN involvement related to pT (higher pT is correlated with higher chance of metastatic SLN, *P* < 0.0001).

		SLN- (%)	SLN = ITC (%)	SLN = pN1mi (%)	SLN = pN1a (%)	Total*
	1mi	41	4	1	0	46
		*89,1*	*8,70*	*2,2*	0	
	1a	63	7	2	1	73
		*86,3*	*9,6*	*2,7*	*1,4*	
	1b	212	6	17	19	254
pT		*83,5*	*2,4*	*6,7*	*7,5*	
	1c	392	25	57	80	554
		*70,8*	*4,5*	*10,3*	*14,4*	
	2	138	17	37	90	282
		*48,9*	*6,0*	*13,1*	*31,9*	
	>2	15	1	1	18	35
		*42,8*	*2,9*	*2,9*	*51,4*	

		861	60	115	208	1244

*The sum of all pT1 cases is 927.

**Table 3:** Number of involved lymph nodes after ALND in case of multiple positive SLNs (*P* < 0.0001).

SLN +	1	2	3	4	5	Total
pN						
pN1a	26	14	1	—	—	41
pN2a	0	7	3	0	0	10
pN3a	0	2	0	1	1	4
						55

**Table 4:** Histological type versus lymph node involvement.

		Histologic type			
	*n* of SLN	IDC %	ILC %	Others*	Total

pT1	0	*37* * 52,1*	12 * 52,2 *	2	51
	1	14 *19,7 *	2 *8,7 *	1	17
	2	7 *9,9 *	3 *13 *	1	11
	>2	13 *18,3 *	6 *25,1 *	1	20

pT2	Tot	71	23		
	0	21 *39,6 *	12 *36,4 *	3	36
	1	17 *32,1 *	6 *18,2 *	0	23
	2	2 * 3,8 *	4 * 12,1 *	0	6
	>2	13 *5 *	11 *23 *	0	24

	Tot	53	33		

*Others: histologic type as mucinous and tubular.

**Table 5:** Axillary recurrences.

		No. PTS	ARA*	ARR	ARM	Total	%
No ALND	SLN-	861	8	3	3	14	1,6%
	SLN = (ITC)	60	1	1	1	3	5,0%
	SLN = pN1mi	20	0	0	0	0	—
	SLN = pN1a	4	0	0	0	0	—
	SLN not id	8	0	0	0	0	—

ALND	SLN = pN1mi	95	0	0	0	0	—
	SLN = pN1a	204	0	0	1	1	0,5%
	FNAC +	239	0	0	1	1	0,4%
	SLN not id	40	0	0	0	0	—
	SLN before 2005	57	0	0	1	1	1,8%

		1588	9	4	7	22	

*ARA: axillary recurrences alone; ARR: axillary recurrences within regional recurrences; ARM: axillary recurrences within metastases.
